# Supplementary material for: Deletion of the protein tyrosine phosphatase PTPN22 for adoptive T cell therapy facilitates CTL effector function but promotes T cell exhaustion
Source: J Immunother Cancer. 2023 Dec 6;11(12):e007614. doi: 10.1136/jitc-2023-007614 (PMC10711921; doi:10.1136/jitc-2023-007614)
Supplement: Supplementary data [file jitc-2023-007614supp001.pdf]

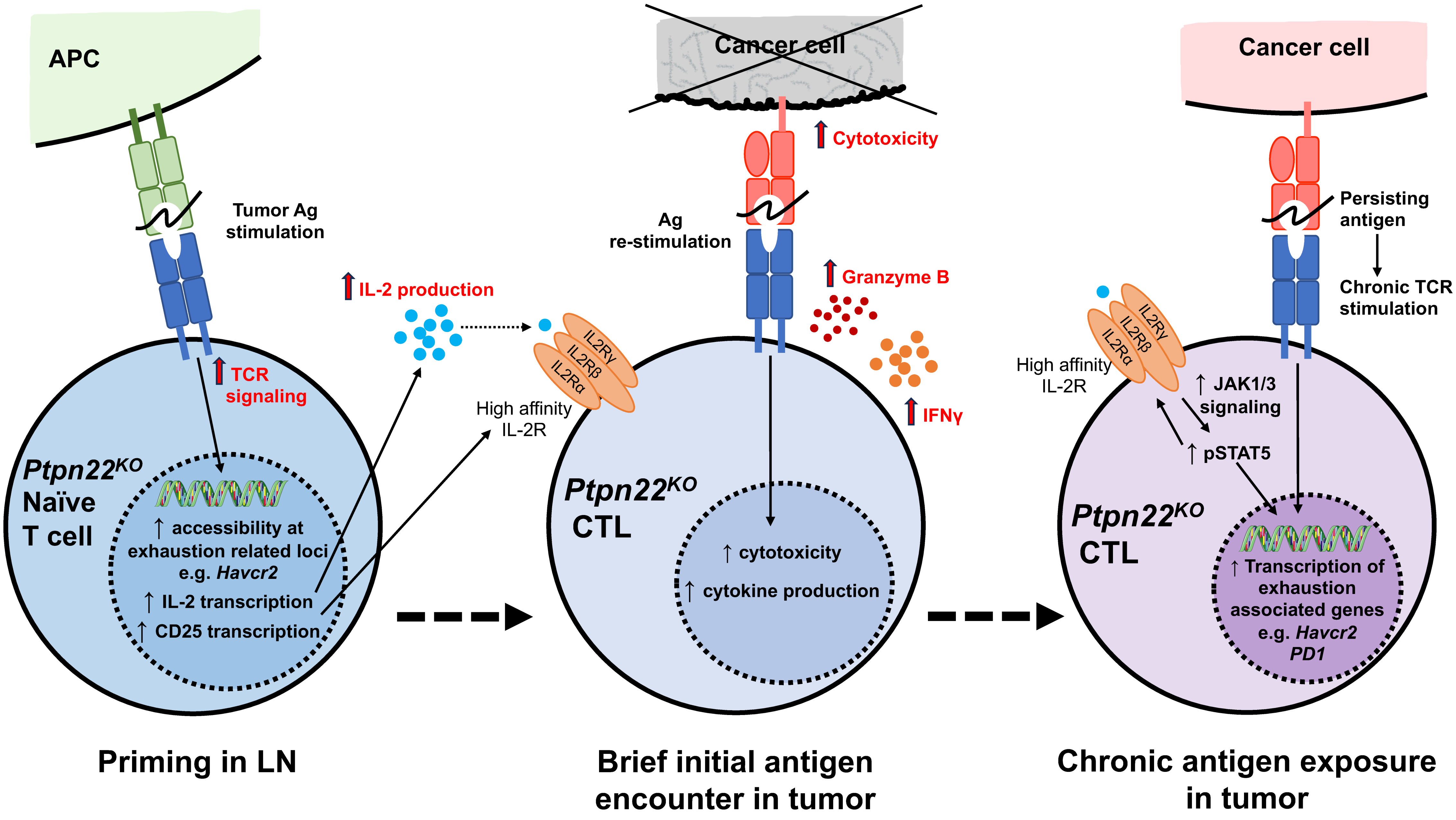
APC
Tumor Ag stimulation
↑ TCR signaling
↑ IL-2 production
High affinity IL-2R
IL2Rγ
IL2Rβ
IL2Rα
Ptpn22KO Naïve T cell
↑ accessibility at exhaustion related loci e.g. Havcr2
↑ IL-2 transcription
↑ CD25 transcription
Cancer cell
↑ Cytotoxicity
Ag re-stimulation
↑ Granzyme B
↑ IFNγ
Ptpn22KO CTL
↑ cytotoxicity
↑ cytokine production
Cancer cell
Persisting antigen
Chronic TCR stimulation
High affinity IL-2R
↑ JAK1/3 signaling
↑ pSTAT5
Ptpn22KO CTL
↑ Transcription of exhaustion associated genes e.g. Havcr2 PD1
Priming in LN
Brief initial antigen encounter in tumor
Chronic antigen exposure in tumor

Deletion of the protein tyrosine phosphatase PTPN22 for adoptive T cell therapy facilitates CTL effector function but promotes T cell exhaustion

Alexandra R. Teagle, Patricia Castro-Sanchez, Rebecca J. Brownlie, Nicola Logan, Simran S. Kapoor, David Wright, Robert J. Salmond, Rose Zamoyska

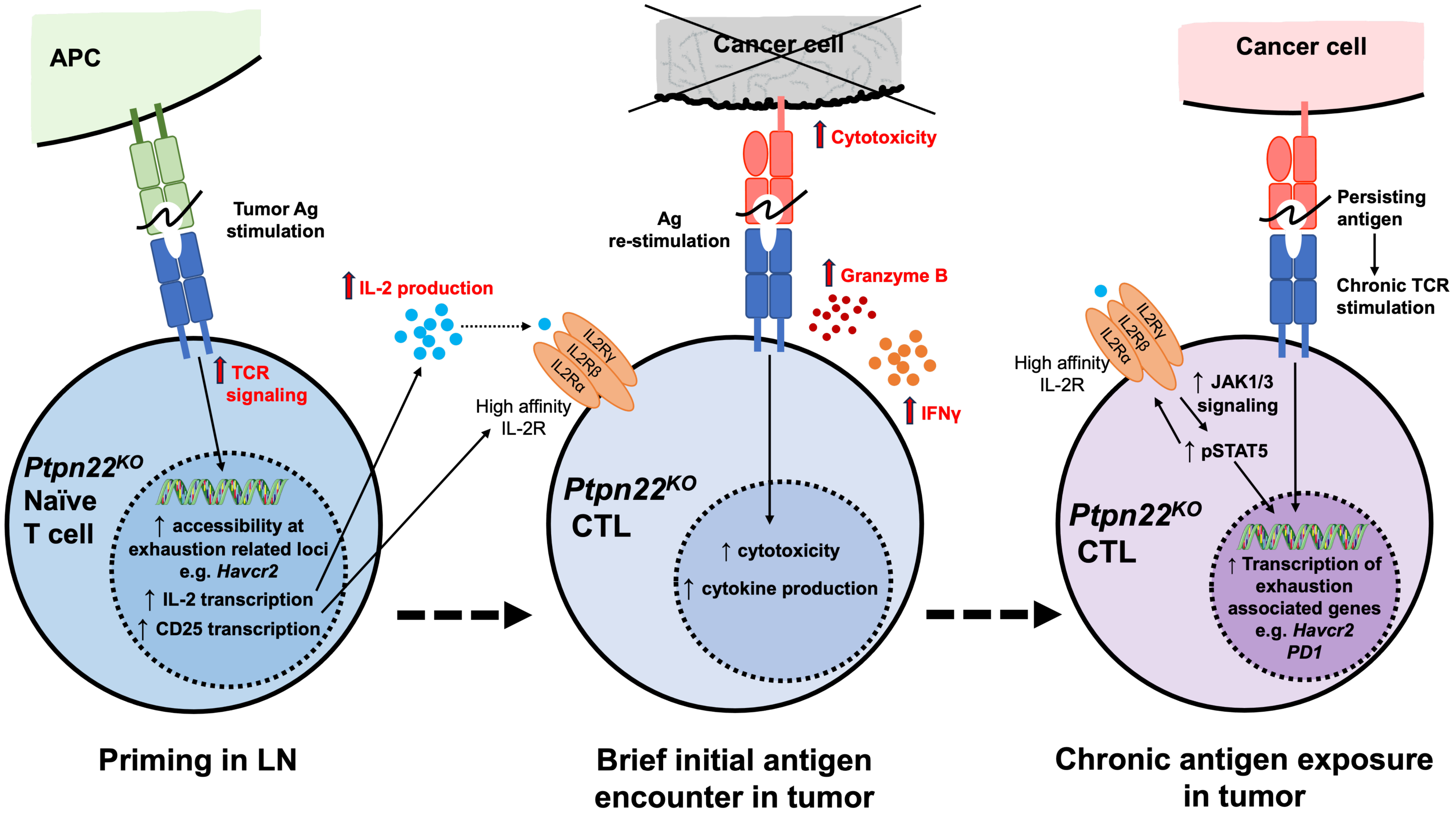

Key points:

- Deletion of PTPN22 in CD8^{+} T cells enhances TCR signalling and improves response to tumour antigen.
- This is associated with enhanced IL-2 responsiveness through increased expression of the high affinity IL-2 receptor alpha chain.
- Where tumour clearance is incomplete and antigen persists, enhanced TCR and IL-2 signals in *Ptpn22*^{KO} T cells lead to more rapid acquisition of an exhausted phenotype, including overexpression of inhibitory receptors such as PD-1 and TIM-3.
